# Preparation and performance evaluation of non-foaming styrene-acrylic latex for cementing slurry

**DOI:** 10.1098/rsos.221319

**Published:** 2023-02-22

**Authors:** Shenglai Guo, Ming Li, Sihe Li, Jiaxin Zhao, Yuhuan Bu, Huajie Liu, Yindong Wang

**Affiliations:** ^1^ Key Laboratory of Unconventional Oil and Gas Development (China University of Petroleum (East China)), Ministry of Education, School of Petroleum Engineering, China University of Petroleum (East China), Qingdao 266580, People's Republic of China; ^2^ Dagang Oilfield Downhole Operation Company, Tianjin 300270, People's Republic of China; ^3^ Institute of Petroleum Engineering, PetroChina Tarim Oilfield Company, Korla 841000, People's Republic of China

**Keywords:** latex, foaming, cementing, cement slurry

## Abstract

The latex used conventionally for oil-well cementing can lead to serious foaming issues in the cement slurry, which not only affects the accurate measurement of the density of the latex-containing cement slurry, but also is detrimental to cementing construction. A large amount of a foam stabilizer used for latex preparation is mainly responsible for foaming of the latex-containing cement slurry. In this study, soap-free emulsion polymerization was conducted using 2-acrylamido-2-methylpropanesulfonic acid (AMPS), styrene (St), and butyl acrylate (BA) as the reaction monomers and the effects of the AMPS dosage, monomer ratio, reaction temperature and stirring speed on the performance of the latex were investigated. The optimum synthesis conditions included a 30% monomer concentration, an St : BA : AMPS monomer ratio of 5 : 4 : 6, a synthesis temperature of 85°C, a stirring speed of 400 r.p.m. and 1.5% of the initiator. As-prepared latex exhibited good filtration loss control, excellent freeze–thaw stability, and extremely low foaming of the cement slurry with the added latex, which was extremely beneficial for on-site cementing construction.

## 1. Introduction

Well cementing is a process in which a casing is inserted into an oil and gas well and a cement slurry is injected into the annulus between the casing and formation [1–3]. The cementing quality of the oil and gas well is related not only to the safe and efficient production of the oil and gas well but also its service life, which is extremely important for the development of the oil and gas field. Since its discovery in 1903, cementing technology has made continuous development, but several problems still exist in oil- and gas-well cementing operations, such as bad mud displacement efficiency, annular gas channelling and cement sheath stress failure, etc. [4–6].

As an important cement slurry additive, latex can effectively alleviate or avoid the issue of gas channelling during the injection of the cement slurry, improve the elastic toughness of the cement stone and prevent the stress failure of the cement sheath during the service process [7–9]. Latex is a particle polymer with a size distribution range of 50–500 nm, which can form a colloidal emulsion by self-dispersion or addition of a surfactant in water; a surfactant is typically added to improve its freeze–thaw stability and prevent abnormal flocculation of the cement slurry [10]. Typically, the types of latex include polyvinyl acetate latex, styrene–butadiene latex, nitrile latex and neoprene latex, etc. [11]. As early as the 1820s, latex was used in Portland cement to improve the performance of cement slurry [12]. After the addition of latex into the cement slurry, latex particles coagulate and form a film adsorbed on the C–S–H surface. Because rubber latex exhibits good flexibility and a high bond strength, the latex cement slurry exhibits several advantages, including improved pumping ability of the cement slurry, reduced permeability of the cement stone, increased tensile strength of cement, reduced shrinkage volume of the slurry and increased elasticity of the cement stone, etc. [10]. In 1957, latex was introduced in oil well cement. In addition to the above advantages, the addition of latex into oil well cement also renders several advantages, such as improved bond performance between cement and the oil well wall, reduced cement sheath cracks during perforating operation, increased resistance capability to pollution of the drilling fluid, reduced slurry filtration amount and improved durability of the cement stone [13].

Although traditional latex can effectively improve various properties of the cement slurry and cement stone, it exhibits a clear disadvantage: a large number of bubbles are produced in the cement slurry, which are difficult to eliminate effectively [14]. A large number of bubbles produced during the preparation of the latex cement slurry affect the accurate density measurement of the cement slurry, which adversely affects the cementing safety and quality. The foaming problem of the latex cement slurry has been investigated in the oil-well cementing industry. Typically, stabilizers in latex are thought to produce a large number of bubbles in the latex cement slurry [15,16]. A large number of bubbles formed by latex in the cement slurry can easily form bubble-rich areas, thereby increasing the cement voids and adversely affecting the density and mechanical properties of the cement slurry [17–19]. For this purpose, a defoaming agent is typically used to reduce bubbles in the cement slurry caused by the added latex; however, a large number of bubbles still cannot be eliminated even after the addition of the defoaming agent [14,20].

The presence of a stabilizer in latex primarily leads to foaming of the latex cement slurry, and the absence of a stabilizer during latex preparation is the main measure to solving the foaming issue of the latex cement slurry. Therefore, in this study, latex is prepared by soap-free emulsion polymerization with no stabilizer introduced, and butyl acrylate (BA), styrene (St) and 2-acrylamido-2-methylpropanesulfonic acid (AMPS) are used as the monomers. The stability of latex and the effect of latex on the fluid loss of the cement slurry are used as the key performance indices. In addition, the latex is characterized by infrared (IR) spectroscopy and transmission electron microscopy (TEM).

## 2. Experimental materials and methods

### 2.1. Experimental materials

St, BA, sodium hydroxide, potassium persulfate and sodium bisulfite were purchased from Sinopharm Chemical Reagent Co., Ltd. AMPS was purchased from Jiangxi Changjiu Agricultural Chemical Co., Ltd. Class G oil well cement was purchased from Sichuan Jiahua Cement Co., Ltd. The dispersant SGJZ was supplied by Shengli Oilfield.

### 2.2. Preparation of latex

A certain amount of AMPS was completely dissolved in deionized water, and the pH of the system was adjusted with NaOH to 5–7. Then, the AMPS solution was transferred to a four-necked flask, and the stirrer and heating switch were switched on. Next, the temperature was increased to 85°C, followed by the addition of St and BA into the solution. After 30 min of continuous stirring, an initiator with 1.5% the mass of the monomer was added (mass ratio of potassium persulfate to sodium bisulfite was 1 : 1). The latex BA-St-AMPS (BSA) was obtained after 2 h of the reaction.

### 2.3. Latex particle size test

First, latex and deionized water were evenly mixed at a mass ratio of 1 : 50, and the mixed liquid was absorbed using a rubber dropper into the sample tube, and the top cover of the sample tube was covered. The particle size was analysed by using a Brookhaven Zeta Potential and Laser Particle Size Analyzer 90Plus Zeta instrument. Each sample was tested in triplicate, and the average value was taken as the particle size of the sample.

### 2.4. Infrared spectral analysis

First, a certain amount of latex BSA was weighed and added into a test tube, followed by the addition of an appropriate amount of anhydrous ethanol and shaking to break the formed emulsion. Second, a certain amount of acetone was added into the solution after breaking the emulsion, followed by high-speed centrifugation and pouring of the supernatant in the test tube. Next, the above steps were repeated thrice, followed by the replacement of acetone with distilled water and repetition of the above steps thrice to completely wash the unpolymerized monomers from the product. Finally, the finished material was placed in an oven for drying at low temperatures. After there was no change in the sample quality, it was removed from the oven and ground into a powder using an agate mortar. Absorption spectra were recorded in the range of 4000–400 cm^−1^ by using a USA Thermo Fisher Nicolet IS50 Fourier transform IR spectrometer.

### 2.5. Transmission electron microscopy analysis

In this experiment, the microscopic morphology of the sample was directly observed on a JEM-2100F field-emission TEM instrument.

### 2.6. Fluid loss test of cement slurry

The cement slurry was prepared using a Waring blender according to the American Petroleum Institute (API) standards [21]. First, latex was added to water, followed by the addition of the class G oil well cement and mixing using a constant speed mixer at a low speed (4000 r.p.m.) for 15 s and then at high speed (12 000 r.p.m.) for 35 s. Second, the mixed cement slurry was poured into a cement slurry filter press and sealed, and the pressure was set to 6.9 MPa. The fluid loss over 30 min was observed. The cement slurry formulation was class G oil well cement + 0.5% SGJZ dispersant + 12.5% latex + 31.5% water.

### 2.7. Latex freeze–thaw stability test

The freeze–thaw stability of latex refers to the stability of the latex system when subjected to alternating freeze–thaw cycles [22]. The freeze–thaw stability of latex is of significance for the production, transport, storage and application of latex. In this experiment, the freeze–thaw stability of latex was tested by taking an appropriate amount of latex in a beaker and freezing it in a refrigerator at −20°C for 18 h. Then, the beaker was removed from the refrigerator and left to thaw at 20°C for 6 h. A complete freeze–thaw cycle was recorded at 24 h. The freeze–thaw test was repeated as mentioned above. The number of freeze–thaw times was recorded. The more the number of freeze–thaw times of latex before the latex loses its stability, the better the freeze–thaw stability of the latex [23].

## 3. Results and discussion

### 3.1. Effect of the AMPS content on latex properties

Herein, AMPS was used instead of traditional latex stabilizers; thus, its dosage is extremely critical to the performance of the latex. In this section, the ability of latex to control fluid loss and appearance of latex at 10%, 20%, 30%, 40% and 50% AMPS dosages were investigated. Table 1 summarizes the obtained data.

**Table 1.** Properties of latex at different AMPS dosages.

no.	AMPS (%)	product appearance
1	10	more white particulate precipitation
2	20	less white particulate precipitation
3	30	bluish emulsion
4	40	bluish emulsion
5	50	bluish emulsion

**Figure 1.**
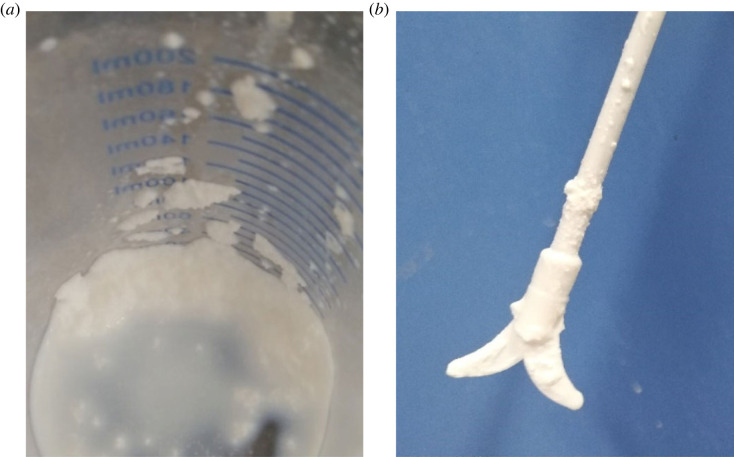
The white precipitates of latex after being synthesized.

The products synthesized by the addition of 10% and 20% AMPS exhibited a large amount of white precipitates (figure 1). It can be seen from figure 1 that a large amount of white solids precipitated at the bottom of the four-necked flask and adhered to the stirring paddle. This result was probably related to the fact that by the addition of a low amount of AMPS, the amount of sulfonic acid groups on the microsphere surface was small, and the resulting microspheres were unstable and prone to aggregation. With the increase in the addition amount of AMPS, the number of sulfonic acid groups on the microsphere surface increased, and the prepared latex exhibited good stability at an AMPS content of greater than 30%.

**Figure 2.**
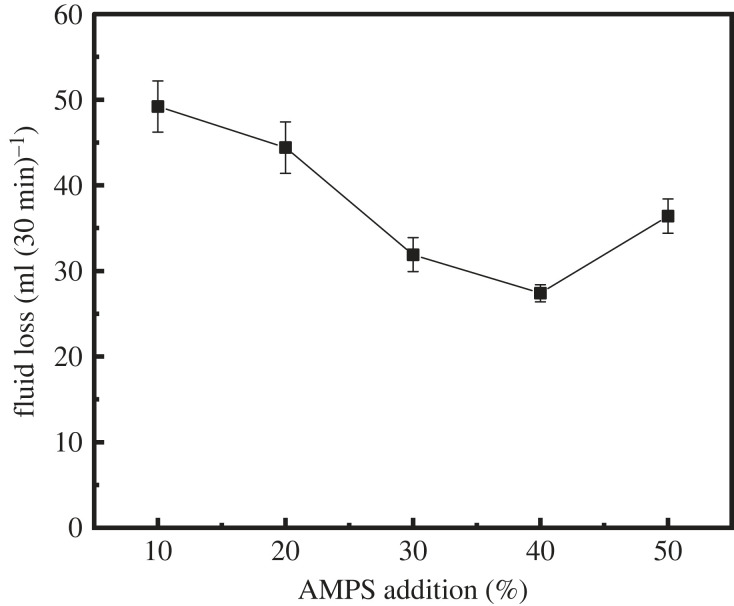
Variation of fluid loss with different AMPS dosages.

**Figure 3.**
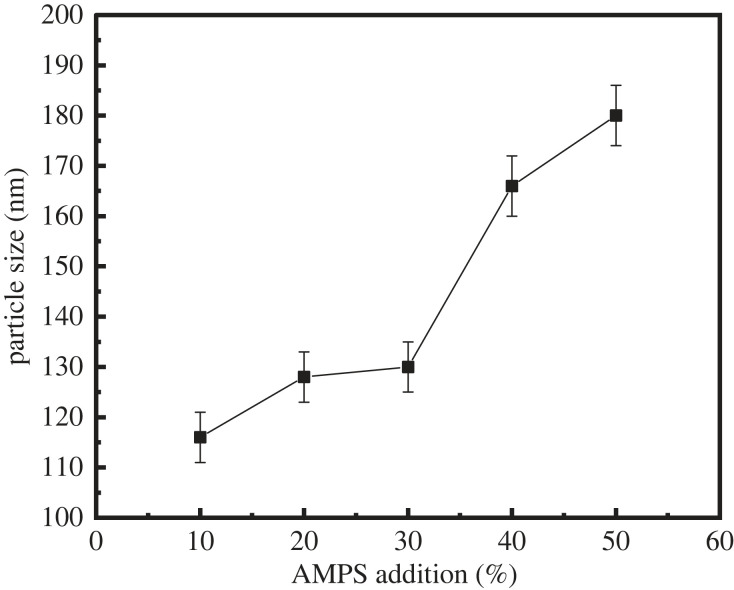
Variation of particle size with AMPS dosages.

Figure 2 shows the effect of the addition of AMPS on the filtration control ability of latex. With the increase in the AMPS dosage, the fluid loss of the cement slurry decreased first and then increased, and the lowest fluid loss was 27.32 ml at an AMPS dosage of 40%. To clarify the effect of the AMPS dosage on the cement slurry fluid loss, the latex particle size was examined under different AMPS dosages (figure 3). With the increase in the AMPS dosage, the latex particle size gradually increased (figure 3). The reasonable increase in the latex particle size can effectively enhance the ability of latex particles to block the pores of cement particles, gradually reducing the fluid loss of the cement slurry. However, the further increase in the latex particle size was not conducive to the free arrangement of latex particles in the pores between cement particles; hence, the fluid loss of the cement slurry slightly increases, but the fluid loss can still be maintained at a low level.

### 3.2. Effect of the monomer ratio on latex properties

**Figure 4.**
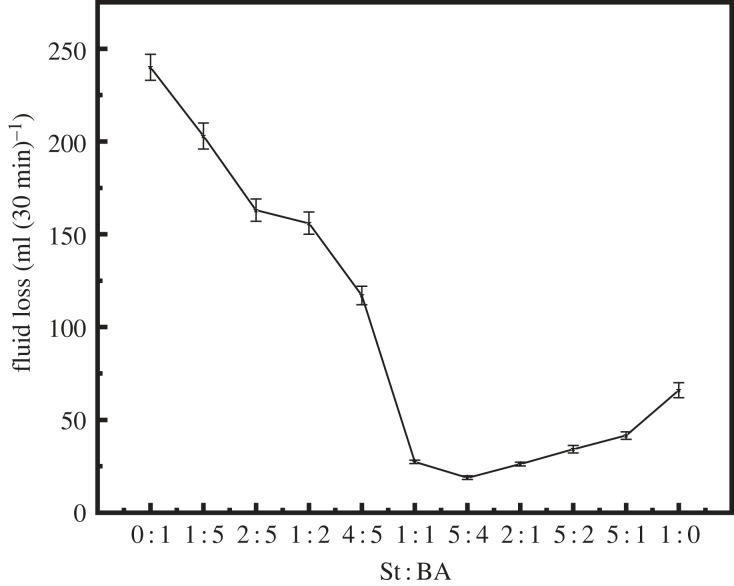
Variation of fluid loss with different ratios of St : BA.

The latex performance was investigated by the addition of 40% AMPS and a monomer St to BA mass ratios of 0 : 1, 1 : 5, 2 : 5, 1 : 2, 4 : 5, 1 : 1, 5 : 4, 2 : 1, 5 : 2, 5 : 1 and 1 : 0. Table 2 and figure 4 show the experimental results.

**Table 2.** Properties of latex with different St : BA ratios.

no.	St : BA (quality ratio)	latex appearance
1	0 : 1	more white precipitation
2	1 : 5	bluish emulsion
3	2 : 5	bluish emulsion
4	1 : 2	bluish emulsion
5	4 : 5	bluish emulsion
6	1 : 1	bluish emulsion
7	5 : 4	bluish emulsion
8	2 : 1	bluish emulsion
9	5 : 2	bluish emulsion
10	5 : 1	less white precipitation
11	1 : 0	more white precipitation

At St to BA mass ratios of 0 : 1, 5 : 1 and 1 : 0, the prepared latex solutions were unstable, with the generation of a certain precipitate, and the latex exhibited better stability under the conditions of the other monomer ratios (table 2). Figure 4 shows the fluid loss of a latex-containing cement slurry as a function of the St to BA mass ratio. On the whole, with the increase in the St ratio, the fluid loss of the cement slurry decreased first and then increased. By the addition of only the soft monomer BA, the maximum fluid loss of the cement slurry occurred in 5 min. By calculation, the fluid loss of the cement slurry was 240 ml (30 min)^−1^. With the increase in the amount of the hard monomer St, the ability of latex to control fluid loss gradually increased, it reached the optimum value at an St to BA mass ratio of 5 : 4, and the fluid loss was only 18.98 ml. With the further increase in the amount of the hard monomer St, the fluid loss of the cement slurry increased gradually. The results revealed that with the addition of a high amount of soft monomer BA, the latex particles were soft, and it was difficult for the particles to bear the sealing pressure; hence, the ability to control fluid loss is poor. With the gradual increase in the amount of the hard monomer St, the content of benzene rings in the molecular structure of the product increased gradually. The presence of benzene rings resulted in the difficult internal rotation of the polymer molecular chain, considerably improving the stiffness of the molecular chain and rendering the ability to withstand a certain pressure; hence, the fluid loss of the cement slurry is well controlled. With the further increase in the addition of the hard monomer St, the latex particles became extremely inflexible, and deformation was difficult during extrusion, making the effective sealing of cement particle pores difficult.

### 3.3. Effect of the synthesis temperature on latex properties

**Figure 5.**
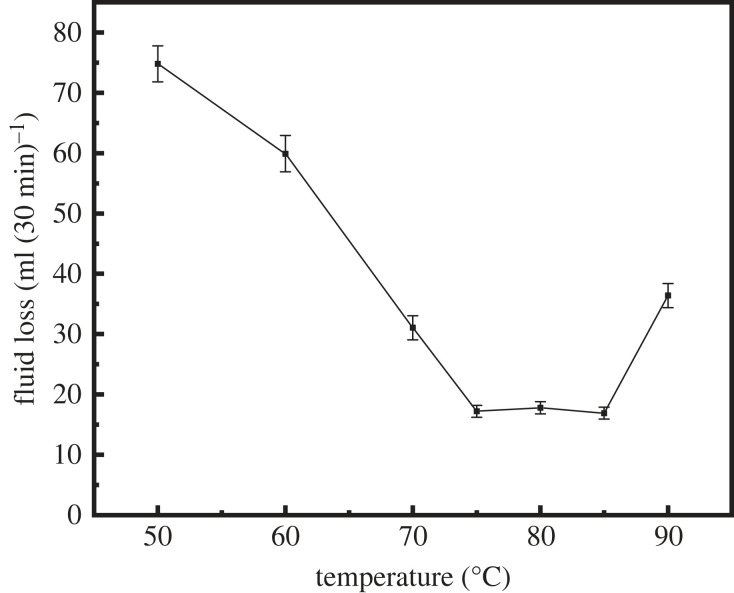
Variation of fluid loss with different synthesis temperatures.

**Figure 6.**
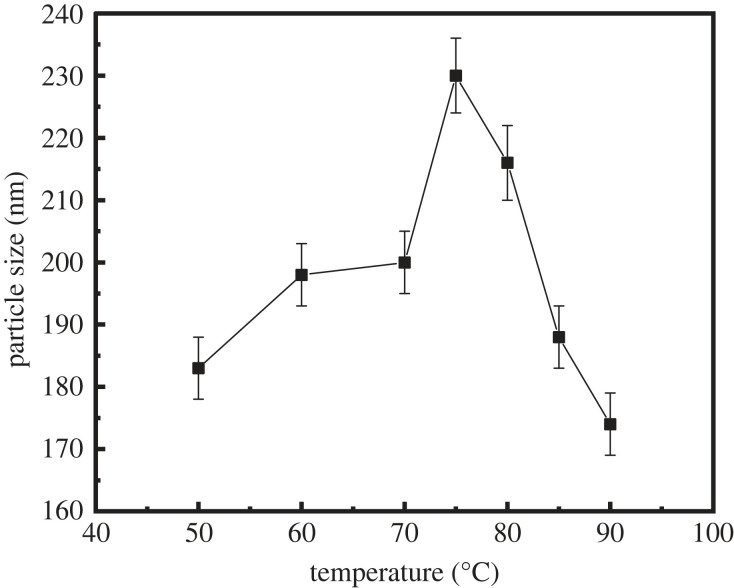
Variation of the particle size of latex with different synthesis temperatures.

In this section, the effect of the synthesis temperature on the properties of latex was investigated. Table 3, figures 5 and 6 show the properties of the latex prepared at reaction temperatures of 40°C, 50°C, 60°C, 70°C, 75°C, 80°C, 85°C and 90°C.

**Table 3.** Appearance of latex at different synthesis temperatures.

no.	synthesis temperature (°C)	latex appearance
1	40	unaggregated
2	50	bluish emulsion
3	60	bluish emulsion
4	70	bluish emulsion
5	75	bluish emulsion
6	80	bluish emulsion
7	85	bluish emulsion
8	90	less white precipitation

As can be observed in table 3, the emulsion did not react at a synthesis temperature of 40°C. At 90°C, a small amount of the white precipitate was generated, and the emulsions prepared at 50–85°C exhibited good stability.

Figure 5 shows the effect of the synthesis temperature on the ability to control the fluid loss of the latex: with the increase in the synthesis temperature, the fluid loss of the cement slurry first decreased and then increased, and the lowest fluid loss of the cement slurry was observed in the temperature range of 75–85°C. Figure 6 shows the effect of the synthesis temperature on the particle size of the latex: the highest effect was observed in the temperature range of 75–85°C. A clear correlation between the fluid loss of the cement slurry and the particle size of latex was observed. Therefore, the optimum latex synthesis temperature range is selected as 75–85°C.

### 3.4. Effect of the stirring speed on latex properties

**Figure 7.**
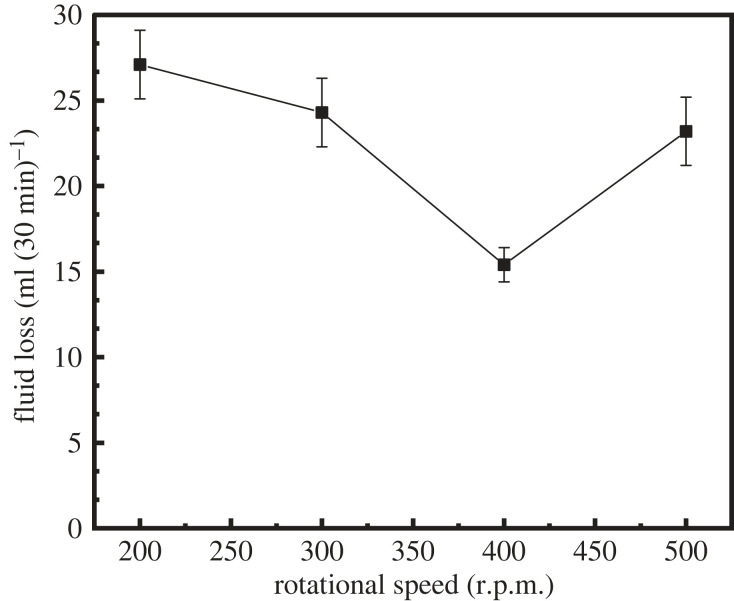
Variation of fluid loss with different rotational speeds.

**Figure 8.**
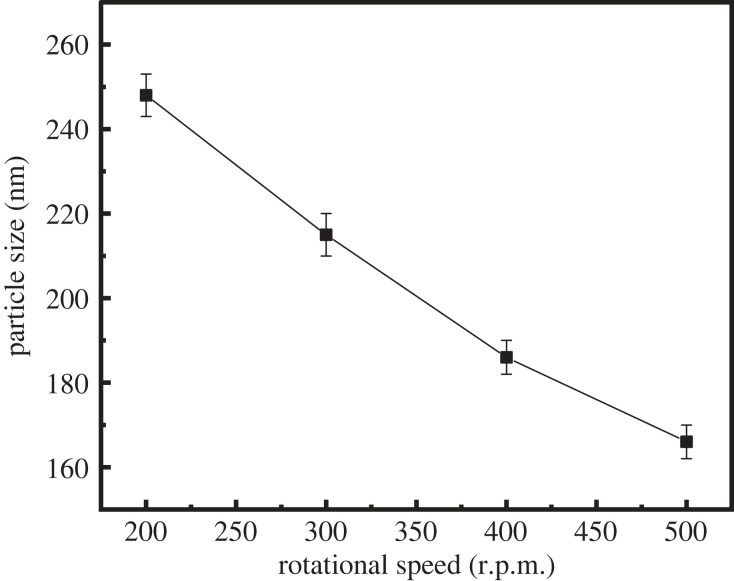
Variation of the particle size of latex with different rotational speeds.

Stirring speed typically exerts a large effect on the particle size and stability of latex [24]. In this section, the effect of the stirring speed on the properties of latex was investigated. Table 4, figures 7 and 8 show the properties of latex under stirring speeds of 200, 300, 400 and 500 r.p.m.

**Table 4.** Appearance of latex under different stirring speeds.

no.	rotational speed (r.p.m.)	latex appearance
1	200	less white precipitation
2	300	less white precipitation
3	400	bluish emulsion
4	500	bluish emulsion

Under stirring speeds of 200 and 300 r.p.m., a small amount of white precipitate was observed in latex, indicative of poor settling stability (table 4). At a rotational speed of greater than 300 r.p.m., the latex exhibited good settling stability. With the increase in the mixing speed, the fluid loss of the cement slurry increased first and then decreased, and the lowest fluid loss of the cement slurry was observed at a mixing speed of 400 r.p.m. (as shown in figure 7). At a stirring speed of less than 400 r.p.m., the fluid loss of the cement slurry was slightly higher, which may be caused by the poor stability of latex. At a stirring speed of greater than 400 r.p.m., the fluid loss of the cement slurry was slightly higher, which may be caused by the decrease in the latex particle size with the increase in the stirring speed (as shown in figure 8).

### 3.5. Infrared spectrum analysis of latex BSA

**Figure 9.**
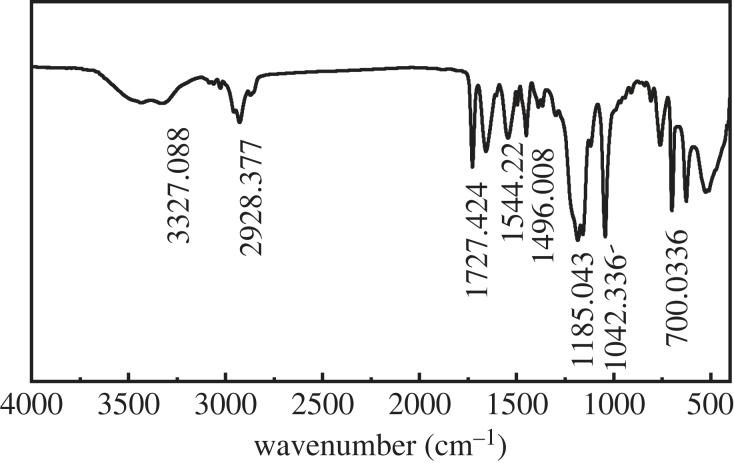
Infrared spectra of BSA latex.

Figure 9 shows the IR spectral analysis results of latex BSA. The stretching vibration absorption peak of -NH in AMPS was observed at 3327.08 cm^−1^, and the stretching vibration absorption peak of -CH in copolymer alkyl was observed at 2928.37 cm^−1^. The absorption peak of C=O in BA was observed at 1727.42 cm^−1^, and the stretching vibration absorption peaks of the benzene ring C=C in styrene were observed at 1544.22 and 1496.00 cm^−1^. The stretching vibration absorption peak of C-O in BA was observed at 1185.04 cm^−1^, and the stretching vibration peak of S=O in AMPS was observed at 1400–800 cm^−1^. The stretching absorption peak of S-O in the copolymer AMPS was observed at 700.03 cm^−1^. Hence, latex is composed of St, BA and AMPS. At the same time, as the absorption peak corresponding to the C=C stretching vibration was in the range of 1675–1640 cm^−1^, no absorption peak was observed in this range, indicating that a compound containing C=C is absent and that all three monomers react to produce latex.

### 3.6. Transmission electron microscopy of latex BSA

**Figure 10.**
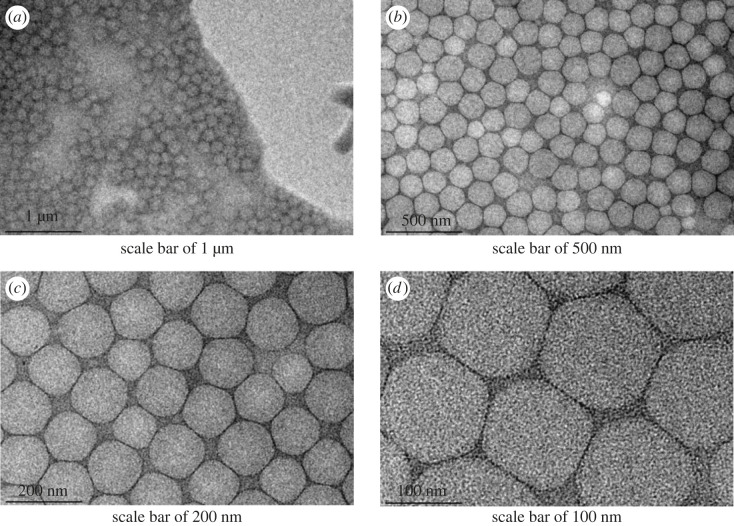
Transmission electron microscopy (TEM) images of BSA. (*a*) Scale bar of 1 µm. (*b*) Scale bar of 500 nm. (*c*) Scale bar of 200 nm. (*d*) Scale bar of 100 nm.

The structure and properties of the sample were observed by TEM analysis. Figure 10 shows the structure of the oil well cement latex BSA particles synthesized in this experiment. The as-synthesized latex particles exhibited a uniform size and good spherical morphology.

### 3.7. Effect of latex on bubbles in the cement slurry

**Figure 11.**
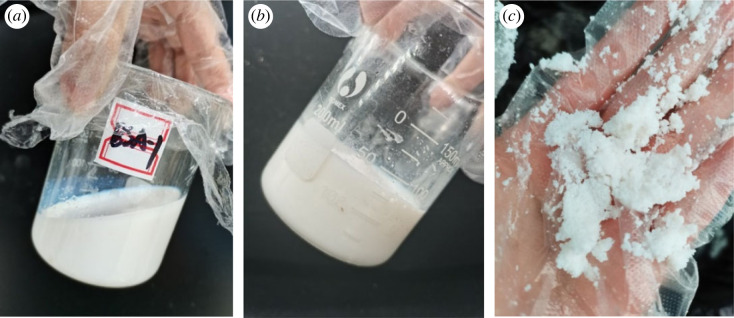
BSA latex (*a*) and styrene–butadiene latex (*b*,*c*).

The freeze–thaw stability of latex refers to the stability of latex during alternating freezing and thawing processes, which is a key index to measure the storage and transportation capacity of latex in winter. The experimental results revealed that the prepared latex BSA could still recover after seven freeze–thaw cycles without precipitation (Figure 11*a*). This result revealed that latex exhibits a good freeze–thaw stability, and it is convenient for transportation, storage, and use at sub-zero temperatures. The contrast sample of styrene–butadiene latex exhibited clear gelation after one freeze–thaw cycle, and it changed into a white solid substance (figure 11*b*) and was crushed easily (figure 11*c*), indicative of the poor freeze–thaw stability of styrene–butadiene latex.

### 3.8. Effect of latex on bubbles in the cement slurry

**Figure 12.**
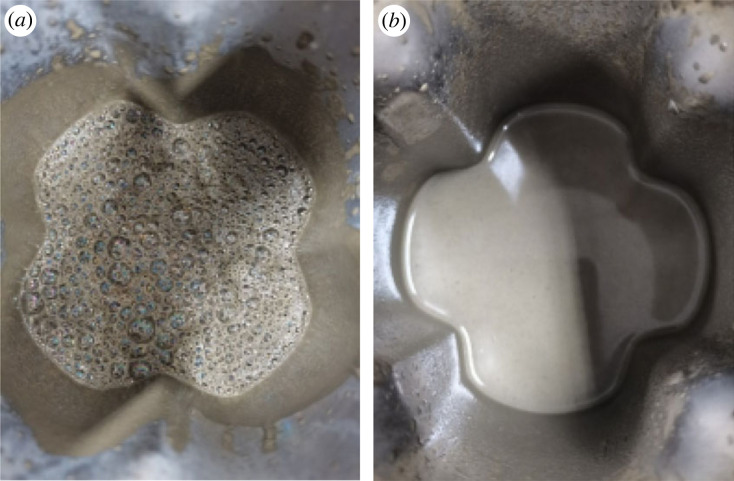
Cement slurry after high-speed mixing. (*a*) Cement slurry with styrene–butadiene latex. (*b*) Cement slurry with BSA latex.

For a conventional cement slurry, it is generally desirable to have as few air bubbles as possible in the prepared slurry to accurately measure the density of the slurry. The addition of cement admixtures often results in the formation of air bubbles in the cement slurry, seriously interfering with the accurate measurement of the cement slurry density. In this section, the effect of the commonly used styrene–butadiene latex in the field was compared and evaluated with the latex prepared herein on the amount of air bubbles produced during the preparation of the cement paste (figure 12). As can be observed from the graph, the cement slurry with styrene–butadiene latex generated a large number of air bubbles after preparation, which were difficult to eliminate even with the addition of a defoaming agent. However, the cement slurry with BSA latex essentially did not generate air bubbles, which was extremely beneficial to the cementing process in the field. The large number of bubbles in the cement slurry with the styrene–butadiene latex was mainly attributed to the large number of surfactants introduced in the styrene–butadiene latex to improve its poor stability. Herein, the latex was prepared by soap-free emulsion polymerization, and the latex exhibited good stability; hence, the addition of a surfactant is not required, and a large number of bubbles are not formed in the cement slurry.

## 4. Conclusion

To prevent considerable foaming of the latex cement slurry, soap-free emulsion polymerization was employed to prepare latex, which did not require a stabilizer to achieve good stability of the latex. Based on the experimental results, the following conclusions can be drawn:

(1) The stability of latex was significantly affected by AMPS addition, with good stability at an AMPS content of greater than 30%. With the increase in the addition amount of AMPS, the fluid loss of the cement slurry decreased and then increased. The analysis suggested that this result may be attributed to the effect of the AMPS addition on the particle size of the latex, resulting in a change in the fluid loss of the cement slurry.
(2) With the increase in the mass ratio of St : BA, the fluid loss of the cement slurry first decreased and then increased. This result indicated that a reasonable ratio of hard and soft monomers helps to ensure that the latex exhibits an ability to control fluid loss.
(3) With the increase in the synthesis temperature, the fluid loss of the cement slurry decreased first and then increased. The synthesis temperature also mainly affected the particle size of latex as well as the fluid loss of the cement slurry.
(4) With the increase in the mixing speed, the fluid loss of the cement slurry increased first and then decreased, and the fluid loss of cement slurry was the lowest at a mixing speed of 400 r.p.m.
(5) BSA latex exhibited good stability after seven freeze–thaw cycles between −20°C and 20°C. In addition, BSA latex did not cause foaming issues in the prepared latex cement slurry.

## Data Availability

The data are available at the Dryad Digital Repository: https://doi.org/10.5061/dryad.bzkh189d2 [25].
